# Flux Control in a Defense Pathway in *Arabidopsis thaliana* Is Robust to Environmental Perturbations and Controls Variation in Adaptive Traits

**DOI:** 10.1534/g3.115.021816

**Published:** 2015-09-10

**Authors:** Carrie F. Olson-Manning, Christopher F. Strock, Thomas Mitchell-Olds

**Affiliations:** * Department of Ecology and Evolution, University of Chicago, Illinois 60637; † Department of Plant Biology, Pennsylvania State University, University Park, Pennsylvania 16802; ‡ Department of Biology, Duke University, North Carolina 27708

**Keywords:** flux control, *Arabidopsis thaliana*, glucosinolate pathway, herbivory

## Abstract

The connections leading from genotype to fitness are not well understood, yet they are crucial for a diverse set of disciplines. Uncovering the general properties of biochemical pathways that influence ecologically important traits is an effective way to understand these connections. Enzyme flux control (or, control over pathway output) is one such pathway property. The flux-controlling enzyme in the antiherbivory aliphatic glucosinolate pathway of *Arabidopsis thaliana* has majority flux control under benign greenhouse conditions and has evidence of nonneutral evolution. However, it is unknown how patterns of flux control may change in different environments, or if insect herbivores respond to differences in pathway flux. We test this, first through genetic manipulation of the loci that code for the aliphatic glucosinolate pathway enzymes under a variety of environments (reduced water, reduced soil nutrients, leaf wounding and methyl jasmonate treatments), and find that flux control is consistently in the first enzyme of the pathway. We also find that a generalist herbivore, *Trichoplusia ni*, modifies its feeding behavior depending on the flux through the glucosinolate pathway. The influence over herbivore behavior combined with the consistency of flux control suggests that genes controlling flux might be repeatedly targeted by natural selection in diverse environments and species.

Understanding the connection between genetic variation and its phenotypic consequences has been a central goal of diverse biological fields, from evolutionary biology to biotechnology. One way to approach the genotype−phenotype connection is through pathway and network modeling, as these approaches provide extensive information on interactions and correlated expression among genes and proteins. However, large-scale functional models derived from dynamic flux analyses or kinetic models are challenging to quantify and time-consuming to validate (van Eunen *et al.* 2012; Antoniewicz 2013), especially in multicellular eukaryotes (Allen *et al.* 2009). In addition, although metabolic models that combine metabolic gene annotations, network topology, and stoichiometry of metabolites have substantial predictive capability (Mintz-Oron *et al.* 2012), these methods still leave gaps in a network where annotation is incomplete (de Oliveira Dal’Molin and Nielsen 2013), restricting the usefulness in applying known changes in gene expression to organism phenotypes and resulting changes in fitness.

However, the observation that enzymes with significant pathway flux control often are encoded by genes with sequence signatures of adaptive evolution supports a functional connection between metabolic variation and whole-organism traits (Flowers *et al.* 2007; Olson-Manning *et al.* 2013; Lavington *et al.* 2014). Genetic studies of *Drosophila* found that enzymes with presumed flux control in glycolysis alter adult flight performance (Eanes *et al.* 2006) and clines in gene frequency are concentrated in glycolytic and pentose shunt enzymes thought to influence flux balance in response to temperature and climate (Lavington *et al.* 2014). For glucosinolates in Arabidopsis, the first biosynthetic step leading to short-chain aliphatic glucosinolates (CYP79F1, Figure 1) has primary flux control and is encoded by the only pathway gene showing clear evidence of non-neutral evolution in that study (Olson-Manning *et al.* 2013). In closely related *Boechera stricta*, the orthologous locus shows accelerated biochemical evolution and controls variation for herbivore damage and plant fitness in nature (Prasad *et al.* 2012). These concordant results across 15 million years of evolutionary divergence (Rushworth *et al.* 2011) support the hypothesis that genes controlling pathway flux might be repeated targets of natural selection due to consistent physiological effects in diverse environments and species.

**Figure 1:**
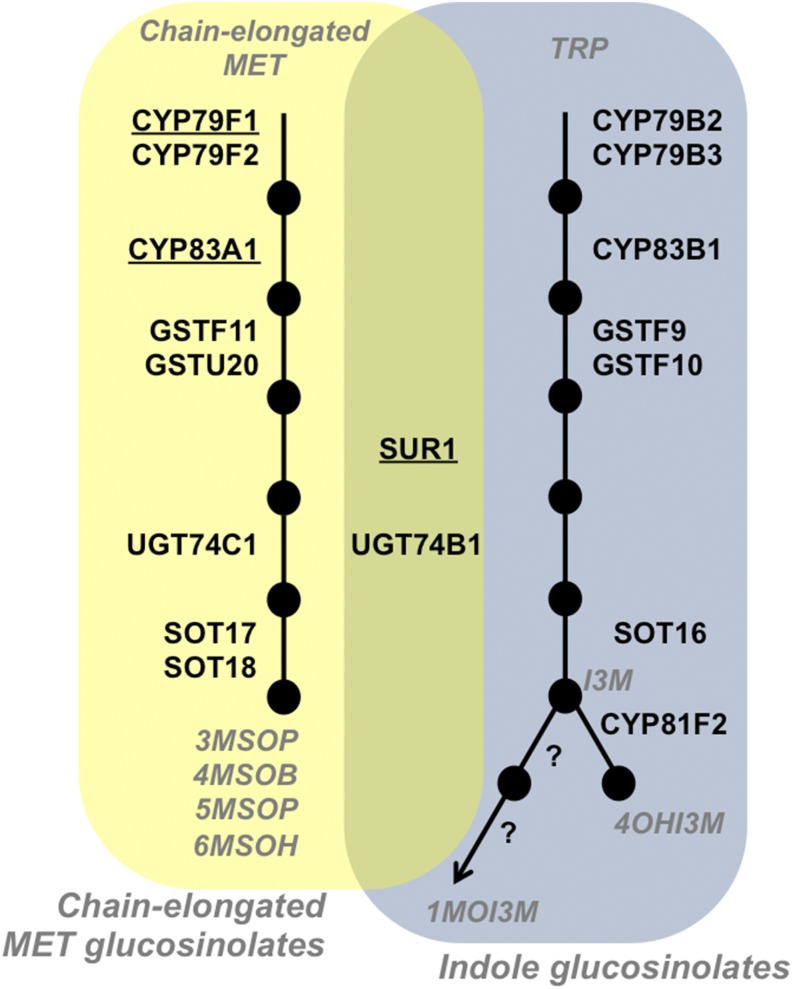
The aliphatic and indolic glucosinolate pathways in *Arabidopsis thaliana*. Aliphatic glucosinolates are synthesized by enzymatic reactions on the left pathway (yellow) beginning with chain-elongated methionine (MET) (Sønderby *et al.* 2010). Indolic glucosinolates are synthesized from tryptophan (TRP) by the right (blue) side of the pathway. Both pathways use the enzymes SUR1 and UGT74B1 found in the green intersection of the pathways. Black circles depict metabolites and are labeled in gray italics where necessary. Black lines connecting metabolites signify enzymatic reactions. Underlined enzymes were those manipulated in this study.

Testing this hypothesis requires two lines of evidence. First, flux control must be robust across a range of growth conditions, so that experimental estimates of flux control apply across different environments. Second, enzymes showing flux control must alter not only metabolite concentrations, but also must impact whole-organism traits that influence ecological fitness.

To test whether environmental conditions alter the distribution of flux control among enzymes in a biochemical pathway, we studied the effects of environmental treatments on the aliphatic glucosinolate pathway (Figure 1) in *Arabidopsis thaliana*. We examined nutrient limitation and water limitation because of their importance for organism growth and glucosinolate concentration (Mailer and Cornish 1987; Bouchereau *et al.* 1996). Likewise, methyl jasmonate (MeJA) and mechanical wounding treatments change the proportion of aliphatic and indolic glucosinolates in some *Brassica* species (Koritsas *et al.* 1991; Bodnaryk 1994). Jasmonate and MeJA are phytohormones that influence a variety of cellular processes including senescence, growth inhibition, and the induction of secondary metabolism (Pauwels *et al.* 2009). Jasmonates also increase the amount of indolic glucosinolates relative to aliphatic glucosinolates (Bodnaryk 1994). Mechanical crushing has similar effects as jasmonate but also increases the expression of both aliphatic and indolic compounds (Koritsas *et al.* 1991). We hypothesized that subjecting plants to these treatments could change the quantity or quality of the glucosinolate profiles produced and might reveal possible changes in pathway flux control.

We chose three genes in the glucosinolate pathways on which to measure flux control under these diverse experimental conditions (underlined in Figure 1). *Cyp79f1* encodes the enzyme responsible for the first step in the pathway, and has majority flux control under typical greenhouse conditions (Olson-Manning *et al.* 2013). Two other genes, *Cyp83a1* and *Sur1*, had not shown significant flux control but have other interesting properties. *Cyp83a1* shows a rate of divergence that is 10 times lower than other genes in the pathway, as expected for a gene under strong purifying selection. It is hypothesized that genes under stabilizing selection with high flux control should exhibit strong indications of purifying selection. Therefore, we hypothesize that *Cyp83a1* may have majority flux control during at least a portion of the life cycle of *A. thaliana*. The SUR1 enzyme functions in both aliphatic and indolic glucosinolate pathways (Sønderby *et al.* 2010). If the SUR1 enzyme is saturated *in vivo* and shows catalytic preference toward either aliphatic or indolic side-chains, then it might divert flux down one of these two pathways under some environmental conditions. Under these conditions, it may control flux. This enzyme also displays some sequence signatures of non-neutral evolution.

Finally, even if flux control changes due to environmental influence, this change may be unimportant unless it alters whole-organism traits that influence ecological fitness. Mauricio and Rausher (1997) showed that herbivore damage reduces fitness of *A. thaliana* in nature when herbivores are present. Further work shows that glucosinolate concentration is under directional selection in the presence of herbivores and under stabilizing selection in the absence of herbivores (Mauricio 1998). We therefore hypothesize that changes in flux will influence the feeding behavior of generalist herbivores which are highly sensitive to the concentration and type of glucosinolates (Schranz *et al.* 2009). Specifically, the herbivore *Trichoplusia ni* is sensitive to the isothiocyanate glucosinolate breakdown products of some aliphatic glucosinolates made by many *A. thaliana* accessions (Lambrix *et al.* 2001). Thus, selective pressure on pathway flux may occur if herbivore feeding is influenced by changes in glucosinolate concentration, acting specifically on the enzymes controlling that flux.

Here, we ask the following questions to address whether flux control in the glucosinolate pathway (and genetic variation in those flux-controlling enzymes) may be important for adaptive traits: Does flux control shift among enzymes in the aliphatic glucosinolate pathway of *A. thaliana* under different growth conditions? Do changes in gene expression from environmental perturbation predict flux control? And, is herbivore damage by a generalist herbivore, *T. ni*, predicted by changes in flux in the glucosinolate pathway?

## Materials and Methods

### Experimental analyses

#### Measurement of control coefficients under environmental treatments:

To determine whether flux control of three genes involved in glucosinolate production is influenced by environment, offspring of parents heterozygous (HET) for a loss-of-function allele for each of three different genes were grown under various environmental conditions. To summarize, we altered the quantity of each enzyme with *A. thaliana* Agrobacterium TDNA insertion lines (Alonso *et al.* 2003) that contain a loss-of-function insertion for a single gene and result in a decrease in the amount of mRNA produced for that gene. The three genes with a loss-of-function insertion studied here were *Cyp79f1*, *Cyp83a1*, and *Sur1*, confirmed in Olson-Manning *et al.* (2013).

Plants of each of the three TDNA lines were subject to all possible combinations of four factors, with control or altered levels of: reduced water availability (W), reduced soil nutrient availability (S), mechanical leaf crushing (C), and MeJA (J) application (detailed further in this section). Treatments were overlapping, with a total of 16 conditions. For each of the 16 environmental conditions, each genetic contrast contained approximately eight wild-type (WT) and 15 HET replicates, producing a total of 368 individuals per insertion line. All environments were randomized within 24 hr of planting and flats were shuffled weekly.

Seeds from each genotype were planted directly on the soil in 16.5-cm^2^ single cell cone-tainers (Stuewe & Sons Inc., Corvallis, OR). Seeds were placed in the dark at 4° for 72 hr after planting to overcome dormancy. Plants were grown under long-day conditions (16-hr light) in a greenhouse at a temperature of 18° for 28 d. At this point, leaf tissue was collected for genotypic and glucosinolate analysis.

Randomized racks of 96 “cone-tainers” were placed in standing water from time of planting to tissue collection. For the low-water treatment, the bottoms of cone-tainers were allowed to dry and then the lower portion of the cone-tainers was sealed with plastic sleeves (fingers cut from nitrile gloves) 8 d before tissue collection. This restricted water access of plants in the low water treatment, while control plants for this treatment still received bottom watering. This procedure enabled greater statistical power than a split-plot experimental design.

Plants requiring MeJA application were separated from the controls during hormone application and sprayed in a separate room. Each plant was misted with approximately 0.45 mL of 1:1000 MeJA (Bodnaryk 1994) (4.6 molar stock; Sigma-Aldrich) 24 hr before tissue collection. The rack containing treated plants was covered with clear plastic wrap for one hour, uncovered and allowed to sit in the open air before being returned to randomized racks.

Seeds grown in standard levels of nutrients were planted in Fafard 4P Mix covered with approximately 1 cm of Sunshine #1 Natural & Organic mix. Seeds grown in low nutrient conditions were planted in soil consisting of one part perlite: two parts sand: two parts Fafard 4P Mix. The low nutrient soil was covered with approximately 1 cm of Sunshine #1 Natural and Organic Mix to allow seeds to germinate and begin growth with minimal mortality.

Mechanical wounding was accomplished 24 hr before tissue collection by crushing a single leaf with a corrugated refrigerator clip. A leaf other than the damaged leaf was collected for glucosinolate analysis.

Twenty-five days after planting a single true leaf was removed for glucosinolate quantification. To determine glucosinolate concentration, each leaf was weighed and stored for 21 days in 2 ml 70% methanol at 4° and then for 7 d at room temperature. Glucosinolate quantification and mutant genotyping were performed as in (Olson-Manning *et al.* 2013).

#### Measurement of leaf area removed by generalist herbivore:

Given the known role of CYP79F1 in flux control, we only analyzed herbivory on *Cyp79f1* genotypes. A single true leaf from 3-wk-old plants was placed on a moist paper towel (to prevent desiccation) in a 2.5-cm Petri dish for 20−24 hr at 23° with one, second instar *T. ni* larvae, (a generalist herbivore). Photographs of each leaf were taken before exposure to *T. ni*, after 3 hr, and again at 24 hr. Plants that had no leaf area removed after 24 hr were discarded from the experiment to avoid unhealthy or molting larvae. The total area consumed by herbivores after 3 hr was calculated using *ImageJ* (National Institutes of Health).

### Statistical analyses

#### Genotype and environment effect on control coefficients:

Analysis of glucosinolates produced by WT and HET plants under different environmental conditions was conducted with multivariate analysis of variance (MANOVA). Glucosinolate concentrations were log transformed to improve normality. MANOVA was performed for each gene in JMP with the concentrations of the seven glucosinolate products (Supporting Information, Table S1) as dependent variables, with genotype and all four environmental contrasts as fixed effects. We performed two types of analyses in which we either used all the WT individuals from all three lines, or compared each WT to the HET from the same line. The results of both of these analyses agreed for the MANOVA and thus we report only the pooled WT analysis. The full model included the interaction of genotype by each environment (E) and all pairwise interactions of environment by environment.

$$\begin{array}{l} \left[3M\;\mathrm{SOP}\right]\left[4M\;\mathrm{SOB}\right]\left[5\text{M}\;\mathrm{SOP}\right]\left[\text{6M}\;\mathrm{SOH}\right]\left[\text{I3M}\right]\left[\text{4OH}\;\mathrm{I3M}\right]\left[\text{1M}\;\mathrm{OI3M}\right]=\text{genotype}+\text{W}+\text{C}+\text{S}+\text{J}+\\ \text{genotype}\times \text{E}\mathrm{+Ε}\text{E}\times \text{E} \end{array}$$

where *genotype × E* indicates four interactions of genotype with environmental treatment, and *E × E* refers to all pairwise interactions of environmental treatments.

When MANOVA was statistically significant for the Wilk’s Lambda test statistic, subsequent univariate analyses were used to test the significance of treatment on each glucosinolate compound, using α = 0.05 (Scheiner 2001). Because none of the genotype by environment interactions was significant in MANOVA, we pooled WT and HET individuals when testing for the main effects of each treatment. The heat maps were generated with the *heatmap* package in R. The proportional amount of change was calculated by the following equation:

(TC−UC)/UC

where *T_C_* is the concentration of glucosinolate in the treated and *U_C_* is the concentration in the untreated samples. For analyses, we pooled environmental treatments for the univariate analysis, reporting the concentration of each glucosinolate type in each genotype, averaged across all environments. Likewise, there were no genotype by environment interactions so all genotypes were pooled within a treatment for the analysis displayed.

Control coefficients were calculated with the relative expression ratios as previously determined (Olson-Manning *et al.* 2013). Bootstrap confidence intervals (CIs) are reported for the ratio of glucosinolate concentration in the HET compared to the WT, resampling 1000 times in Python (code available from Olson-Manning).

#### Leaf area removed:

Finally, we used two-way analysis of variance to test whether the random effect of flat or the fixed effect of genotype of *Cyp79f1* predicted the absolute amount of leaf area that the herbivore *T. ni* removed after 3 hr from a single leaf.

LR=genotype+flat+genotype×flat

Where *LR* is the amount of leaf area removed after 3 hr.

#### Gene expression meta-analysis:

We used available data for a meta-analysis of the effect of MeJA on the expression of genes in the aliphatic and indolic glucosinolate pathways. Data were harvested from the EMBL Gene Expression Atlas, experiment numbers E-GEOD-17464 (unpublished), and E-GEOD-18667 (unpublished), and E-MEXP-883 (Dombrecht *et al.* 2007). Each of these experiments was performed with the Affymetrix GeneChip Platform on rosette leaf tissue with three biological replicates per treatment on either *A. thaliana* Landsberg or Col-0 genotypes. The treatments were for variable amounts of MeJA (between 0.1 and 50 μM) and different incubation times (1−10 hr), hence results should be interpreted in a qualitative, rather than quantitative manner. Proportional change in expression was calculated from the difference in median value of treated and control samples, divided by the control expression levels. These and proportional changes were averaged among the experiments. Negative values indicated a decrease in expression of the treated compared to the untreated plants.

### Data availability

Knockout lines were obtained through TAIR and are available from the authors upon request.

## Results

We measured glucosinolate concentration in *A. thaliana* on plants that were either HET for gene insertion lines, or homozygous WT. Plants were subjected to four treatments and glucosinolate concentration was measured on 3-wk-old rosette leaves. The overall effects of genotype and environments on concentration were analyzed using MANOVA (Table 1), with genotype (HET *vs.* WT) and the four environments [low water (W), leaf crushing (C), low nutrient soil (S) and MeJA (J) treatments] contrasted to the control treatment (standard growth conditions). In the full model, the interactions of genotype by environment and environment by environment were also tested (Table 1).

**Table 1:** MANOVA effect of environments and enzyme-activity mutants (in each column) on glucosinolate concentrations

	*Cyp79f1* *P*-Value	*Cyp83a1* *P*-Value	*Sur1* *P*-Value
Genotype	1.37 × 10^−20^ ***	0.0237 *	0.0216 *
W	5.14 × 10^−7^ ***	0.0008**	8.08 × 10^−5^***
C	1.11 × 10^−7^***	6.82 × 10^−7^***	4.74 × 10^−5^***
S	4.90 × 10^−8^***	3.73 × 10^−5^***	9.57 × 10^−8^***
J	3.87 × 10^−11^***	7.46 × 10^−14^***	2.79 × 10^−11^***
Geno × W	0.9397	0.8735	0.7093
Geno × C	0.3199	0.4382	0.7466
Geno × S	0.8302	0.3913	0.1797
Geno × J	0.8355	0.5039	0.935
W × C	0.1989	0.4219	0.9822
W × S	0.083	0.0376 *	0.1614
W × J	0.3688	0.7558	0.9404
C × S	0.1257	0.297	0.1088
C × J	0.0538	1.22x10^−5^***	0.0002**
S × J	0.4732	0.5571	0.8802

MANOVA, multivariate analysis of variance.

* *P* < 0.05, **P < 0.01, ***P < 0.001.

For all genes tested, MANOVA showed that genotype and each of the four environments had statistically significant effects on glucosinolate concentrations (Table 1). None of the genotype by environment interactions are significant. Several of the environment-by-environment interactions were significant, but the majority of these interactions were not. Most of the interaction terms were in the C × J, which involve similar pathways. Overall, Cyp79f1 showed robust flux control across all environments, whereas *Cyp83a1* and *Sur1* had marginally significant effects attributable to the large sample size in this experiment.

**Figure 2:**
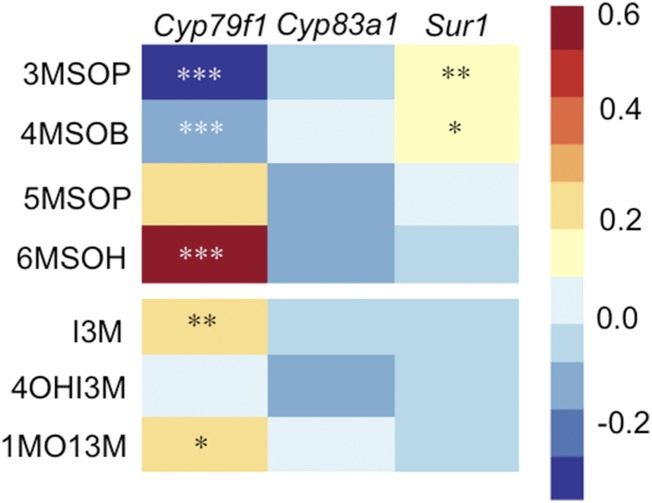
Heatmap of the univariate estimates of the proportional change in glucosinolate concentration of the heterozygous (HET) compared with the wild-type (WT). The four aliphatic glucosinolates are on top (3MSOP, 4MSOB,5MSOP,6MSOH) and the three indolic glucosinolates below (I3M, 4OHI3M, 1MOI3M). Cool colors indicate a decrease in concentration in the HET compared with the WT and warm colors indicate an increase. Asterisks indicate level of significance of the log-transformed data. *** *P* < 0.001, **P < 0.01, *P < 0.05.

We performed univariate tests to examine the effect of genotype on concentration of each glucosinolate compound. Because none of the genotype by environment interactions approached significance in MANOVA (Table 1), we pooled environmental treatments for the univariate analysis, reporting the concentration of each glucosinolate type in each genotype, averaged across all environments. The effect of genotype is highly significant for *Cyp79f1*, as we found previously (Olson-Manning *et al.* 2013). The univariate analyses show that *Cyp79f1* has a much greater effect on glucosinolate concentration than either of the other genes (Figure 2 and Table S2). Aliphatic glucosinolates 3MSOP and 4MSOB (glucosinolate abbreviation key Table S1) were significantly decreased in the HET compared with the WT [as previously reported, (Olson-Manning *et al.* 2013)], but 6MSOH and the indolic glucosinolates I3M and 1MOI3M were increased. *Cyp83a1* and *Sur1* also have significant genotype effects in the MANOVA, but the changes are of much lower magnitude than *Cyp79f1*. None of the univariate comparisons were significant for *Cyp83a1*, but *Sur1* had a significant increase in 3MSOP and 4MSOB in the HET genotype.

We calculated flux control coefficients (Table 2) on the pooled environmental treatment and used the relative expression ratios previously estimated for *Cyp79f1*, *Cyp83a1*, and *Sur1* (Olson-Manning *et al.* 2013). The ratio of HET to WT expression and CIs were calculated by bootstrapping for 1000 iterations. We find results qualitatively similar to our previous study. CYP79F1 has majority flux control for the compounds 3MSOP (λ = 1.118, CI = 1.006−1.247) and 4MSOB (λ=0.740, CI = 0.557−1.018), but not for any other glucosinolate. The other enzymes have much lower estimated control coefficients (λ < 0.47).

**Table 2:** Estimated flux control coefficients calculated using all three lines for WT individuals and 95% confidence interval after 1000 bootstrapping runs

	CYP79F1	CYP83A1	SUR1
3MSOP	1.118 (1.006−1.247)	0.205 (0.147−0.316)	0.006 (-0.014–0.031)
4MSOB	0.740 (0.557−1.018)	0.328 (0.186−0.626)	0.052 (0.032−0.075)
5MSOP	0.040 (0.010−0.076)	0.203 (0.168−0.241)	0.067 (0.047−0.088)
6MSOH	0.061 (0.012−0.125)	0.449 (0.305−0.814)	0.180 (0.139−0.232)
I3M	0.087 (0.047−0.133)	0.461 (0.313−0.720)	0.112 (0.087−0.139)
4OHI3M	0.129 (0.091−0.172)	0.342 (0.264−0.460)	0.112 (0.087−0.141)
1MOI3M	0.200 (0.151−0.257)	0.372 (0.295−0.494)	0.251 (0.214−0.293)

WT, wild type.

**Figure 3:**
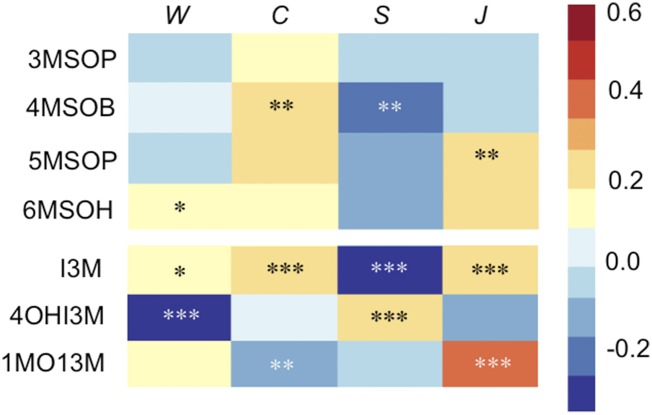
Heat map of the direction of glucosinolate concentration change for the aliphatic and indolic glucosinolate products. The proportional change in glucosinolate concentration of the seven glucosinolate products (abbreviations on the left) following four environmental treatments (W = water deprivation, C = leaf crushing, S = soil nutrient deprivation, J= Methyl Jasmonate treatment). Colors, *P* -value indicators, and scale are identical to Figure 2.

Each of the environmental treatments had a highly significant effect on the quantity and spectrum of glucosinolates produced (Figure 3 and Table S3). However, there are no significant genotype by treatment interactions, indicating the response to environmental treatment is homogenous in the HET and WT.

There were no significant differences in the glucosinolate profiles among the different WT TDNA insert lines, so we pooled all WT lines for our estimates of environmental influences on glucosinolate concentration. Figure 3 and Table S3 show the directions of glucosinolate concentration changes as a result of the four treatments.

**Figure 4:**
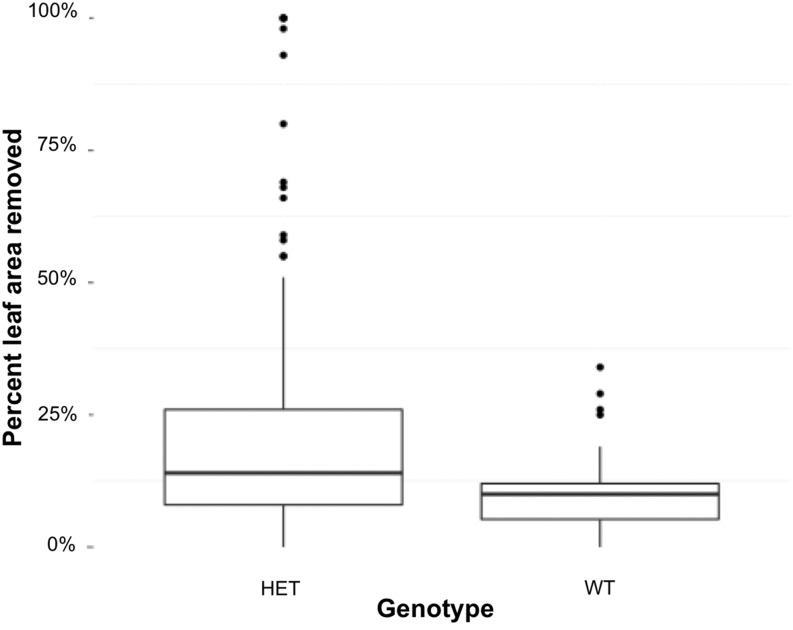
*Trichoplusia ni* removed more leaf area from heterozygous (HET) *Cyp79f1* genotypes than wild-type (WT) *Cyp79f1* genotypes (Two-way analysis of variance *P* = 0.0014). Percent leaf area removed after 3 hr of exposure of a single leaf to a single *T. ni* larvae.

The effect of *Cyp79f1* genotype on the amount of leaf area removed by *T.ni* larvae (Figure 4) was highly significant (*P* = 0.0014) when analyzed with two-way analysis of variance. The HET lines had significantly more leaf area removed than WT lines. We found no significant variation among flats, nor did we find significant flat by genotype interaction influencing the amount of leaf tissue removed.

**Figure 5:**
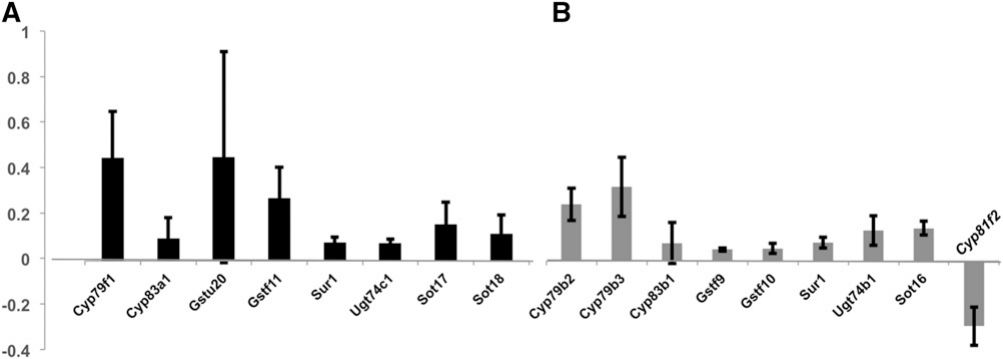
All glucosinolates pathway genes are up-regulated after MeJA treatmeant except for *Cyp81f2*. Average relative expression of genes in the (A) aliphatic (black) and (B) indolic (grey) glucosinolate pathways after MeJA application. Bars are the mean of three different EMBL Gene Expression Atlas experiments and horizontal lines represent standard error. All genes were significantly different from controls in at least two of three experiments.

To determine if expression of glucosinolate pathway genes changed following MeJA treatment, we performed a meta-analysis with data available from three experiments on the EMBL Gene Expression Atlas (Figure 5). All of the genes in the aliphatic and indolic pathways were significantly up-regulated in at least two of the three experiments after MeJA application, except *Cyp81f2*. *Cyp81f2* was significantly down-regulated in all experiments. We checked for expression changes of transcription factors important for aliphatic and indolic gene expression after MeJA application, but missing data from the EMBL on these genes experiments prevents a full analysis.

## Discussion

### Flux control is robust across environments

We find robust control of flux in this ecologically important pathway across a range of environmental conditions that alter biosynthesis of defensive metabolites. Flux control in the aliphatic glucosinolate pathway is consistently in the first enzyme in the pathway, CYP79F1. With a substantial sample size in this study, we also detected minor flux control for CYP83A1 and SUR1, but their influence on overall flux is much less than that caused by CYP79F1. For linear pathways, this is expected (Kacser and Burns 1981) because the presence of an enzyme with high control means other enzymes in the pathway have less control. In branching pathways, several key branch enzymes can exhibit flux control (Rausher 2012). However, no other glucosinolate enzymes tested in this or our previous study (Olson-Manning *et al.* 2013) control flux. We find that CYP79F1 has much greater control coefficients for two aliphatic compounds (3MSOP and 4MSOB) than either CYP83A1 or SUR1. We also find that no enzymes tested have majority control over the other glucosinolates, suggesting that either the flux is distributed among all enzymes that contribute to their synthesis, or the flux-controlling enzyme has not been tested in these experiments (Olson-Manning *et al.* 2013; this study), or might be contained in a connecting pathway.

### Herbivores are sensitive to differences in flux

Glucosinolate concentration influences herbivore pressure in nature (Mauricio and Rausher 1997; Mauricio 1998) and there are numerous examples of glucosinolate profiles changing herbivory (Hopkins *et al.* 2009). We tested whether a generalist herbivore alters its feeding behavior in response to genetic changes in the flux-controlling enzyme in the glucosinolate pathway, and find that the generalist herbivore *T. ni* responds strongly to changes in glucosinolate concentrations in the *Cyp79f1* heterozygote. Significantly more leaf area was removed from the HET *Cyp79f1* than WT plants.

As flux control remains with this enzyme under the majority of environmental conditions tested here, we conclude that generalist herbivores can impose strong selection for CYP79F1 function across a range of environments. This may help explain the evidence we find for non-neutral evolution in the sequence of this gene in *A. thaliana* (Olson-Manning *et al.* 2013), the positive selection detected in the *B. stricta* homolog of *Cyp79f1* in natural populations (Prasad *et al.* 2012), and gives credibility to the often-made assumption that flux control in biochemical pathways is stable over evolutionary time.

### Complexities of flux control

Although we find that HET *Cyp79f1* produce less short-chain aliphatic glucosinolates (3MSOP and 4MSOB), surprisingly, this genotype also has significantly increased quantities of long-chain aliphatic glucosinolates (5MSOP and 6MSOH) (Figure 2). This finding is consistent with known function for synthesis of precursors: before the MET-derived products reach the core glucosinolate pathway in the cytosol, they go through chain elongation in the chloroplast (Sønderby *et al.* 2010). If MET goes through the chain-elongation pathway twice, 3MSOP is produced. If MET goes through three times, then 4MSOP is produced; each subsequent round elongates the chain by another carbon. An abundance of short-chain precursors in the *Cyp79f1* HETs that are not immediately used by the core glucosinolate pathway may incur additional processing in the chain-elongation pathway before entering the core glucosinolate pathway.

These results correspond with the known substrate preferences of CYP79F1 and CYP79F2: CYP79F2 (which has WT function in these genotypes) only metabolizes long-chain methionine derivatives (Chen *et al.* 2003), so elevated levels of long-chain aliphatic glucosinolates are expected in HETs, where the relative activity of CYP79F2 is expected to increase. The result of excess long-chain MET-derived glucosinolates in the *Cyp79f1* HETs is consistent with additional processing in the chain-elongation steps.

*Cyp79f1* HETs produce more indole glucosinolate than the WT, and, to a lesser extent, *Sur1* HETs produce more short-chain aliphatic glucosinolate than the WT (Figure 2) suggesting extensive crosstalk between the indolic and aliphatic pathways. To explore this idea, we first considered the transcription factors known to influence both these pathways. The indolic and aliphatic pathways are regulated by different transcription factors in the Myb protein family; yet, the Mybs affect each other and the same environmental cues (sulfur deficiency, wounding and MeJA) act on both sets of Mybs in complex ways (Gigolashvili *et al.* 2009; Frerigmann and Gigolashvili 2014) and we did not find a coherent explanation to explain our differences in metabolites based on the effect of Mybs. It is also possible that the expression level of one enzyme could influence the expression of different genes in the pathway (Gerosa and Sauer 2011). For example, our results could be explained if knocking down expression of *Cyp79f1* increases expression at *Sur1*. However, further studies are needed that include knockout of the indole pathway genes to fully understand how perturbing gene expression influences crosstalk between these pathways.

#### Genotype-by-environment interactions:

If the level of flux control at a given step changes based on environmental condition, this would cause a significant genotype by environment interaction. We found no evidence for such interactions in the MANOVA (Table 1). Flux control remains with CYP79F1 in all environmental treatments, and the magnitude of the change in glucosinolate concentration is always strongest in *Cyp79f1* HETs (Figure 2). Under more severe conditions, or conditions found in nature, it is possible that predominant flux control might change among these enzymes.

However, the molecular signature of positive selection on *Cyp79f1* [and on *BCMA*, its ortholog in its close relative *B. stricta* (Prasad *et al.* 2012)] is consistent with this enzyme having majority control under most conditions, suggesting that flux control lies at CYP79F1 in a broad range of biologically relevant conditions during a 15-million-yr period (Rushworth *et al.* 2011), which is consistent with theory that control over flux should be primarily in the first enzyme of the pathway and once an enzyme gains control, the enzyme is more likely to retain control (Wright and Rausher 2010).

#### Gene expression and glucosinolate profile:

In three of the four environments tested, the indolic compounds I3M and 1MOI3M are positively correlated, but 4OHI3M changes in the opposite direction (Figure 3). This effect is particularly strong in the MeJA treatment. Using the EMBL Gene Expression Atlas we harvested data from three microarray experiments conducted on the Affymetrix GeneChip Platform that measured gene expression in *A. thaliana* after MeJA treatment. These experiments find that most genes in the aliphatic and indolic glucosinolate pathways increase in expression, consistent with the nearly ubiquitous increase in glucosinolate concentration in our MeJA-treated plants.

One notable exception is *Cyp81f2*, which decreases its expression after MeJA treatment. I3M is converted to 4OHI3M by the enzyme CYP81F2 (Pfalz *et al.* 2009). If CYP81F2 was down-regulated, we would predict to get proportionally less 4OHI3M than 1MOI3M and the reverse if it is up-regulated. MeJA treatment causes reduced *Cyp81F2* expression, and we find a corresponding decrease in 4OHI3M and an increase in I3M and 1MOI3M, as predicted by known biochemical function. The concentration of 4OHI3M is important for resistance to the green peach aphid but has no effect on lepidopteron larvae (Pfalz *et al.* 2009). Furthermore, 4OHI3M is up-regulated in response to fungal attack and a downstream modification of this compound confers broad spectrum antifungal defense for *A. thaliana* (Bednarek *et al.* 2009). Although not tested here, it is possible that CYP81F2 has majority control over the expression of the proportion of indolic compounds under some conditions and thus, fine-tuning of the specific glucosinolate profile is possible and would be selectively advantageous under some environments.

Our results suggest that flux control is robust under a variety of environmental conditions that alter glucosinolate concentrations. If these results are general, evolutionary responses to natural selection may be focused on one, or a few, enzyme-encoding genes, bringing some degree of predictability to metabolic modeling and adaptive changes within a pathway. Further downstream, we found that biochemical constraints on indole glucosinolates are modulated by regulatory changes. Under the conditions tested, the proportion of the different indolic glucosinolates appears to be correlated with the expression of a gene late in the pathway, *Cyp81f2*. Thus fine-tuning of the specific glucosinolate profile under different conditions may depend on modifier genes experiencing other selection pressures.

Reduced activity of CYP79F1, the enzyme with predominant flux control, decreases resistance to herbivory, an ecologically important trait with clear consequences for plant fitness (Mauricio and Rausher 1997; Prasad *et al.* 2012). Therefore, analysis of flux control predicts variation in whole-organism phenotypes, and shows that pathway function constrains adaptive evolution of complex traits. Furthermore, these results could be of value for some agricultural traits. If a single enzyme consistently controls flux through an important pathway under most environmental conditions, then breeding and engineering efforts can be targeted more effectively.

## 

## Supplementary Material

Supporting Information
