# Supplementary material for: Flux Control in a Defense Pathway in Arabidopsis thaliana Is Robust to Environmental Perturbations and Controls Variation in Adaptive Traits
Source: G3 (Bethesda). 2015 Sep 10;5(11):2421–7. doi: 10.1534/g3.115.021816 (PMC4632061; doi:10.1534/g3.115.021816)
Supplement: Supporting Information [file supp_5_11_2421__index.html]

Flux Control in a Defense Pathway in Arabidopsis thaliana Is Robust to Environmental Perturbations and Controls Variation in Adaptive Traits — Supporting Information 

# Flux Control in a Defense Pathway in *Arabidopsis thaliana* Is Robust to Environmental Perturbations and Controls Variation in Adaptive Traits

## Supporting Information for Olson-Manning, Strock, and Mitchell-Olds, 2015

**Files in this Data Supplement:**

- Supporting Information - Tables S1-S3 (PDF, 84 KB)
- Table S1 - Glucosinolate compounds examined in this study, with abbreviations and amino acid precursors. (PDF, 54 KB)
- Table S2 - Univariate estimates of the effect of genotype on glucosinolate concentration. (PDF, 66 KB)
- Table S3 - Univariate estimates of the effect of environmental treatments on glucosinolate concentration. (PDF, 66 KB)
