# Supplementary material for: Flux Control in a Defense Pathway in Arabidopsis thaliana Is Robust to Environmental Perturbations and Controls Variation in Adaptive Traits
Source: G3 (Bethesda). 2015 Sep 10;5(11):2421–7. doi: 10.1534/g3.115.021816 (PMC4632061; doi:10.1534/g3.115.021816)
Supplement: Supporting Information [file supp_g3.115.021816_021816SI.pdf]

G3 · Genes | Genomes | Genetics

Flux control in a defense pathway in *Arabidopsis thaliana* is robust to environmental perturbations and controls variation in adaptive traits

Carrie F. Olson-Manning*, Christopher F. Strock§ and Thomas Mitchell-Olds†

* Department of Ecology and Evolution, University of Chicago, IL 60637, USA

§ Department of Plant Biology, Pennsylvania State University, University Park, PA 16802, USA

† Department of Biology, Duke University, NC 27708, USA

Corresponding author

Carrie F. Olson-Manning, Ph.D.

Department of Ecology and Evolution, University of Chicago, GCIS W504, Chicago, IL 60637, USA

773-834-4664

colsonmanning@uchicago.edu

DOI: 10.1534/g3.115.021816

Table S1 Glucosinolate compounds examined in this study, with abbreviations and amino acid precursors.

Abbreviation	Glucosinolate	Amino acid precursor
3MSOP	3-methylsulfinylpropyl	Homomethionine
4MSOB	4-methylsulfinylbutyl	Dihomomethionine
5MSOP	5-methulsulfinylpentyl	Trihomomethionine
6MSOH	5-methylsulfinylhexyl	Tetrahomomethionine
I3M	Indolylmethyl	Tryptophan
4OHI3M	4-hydroxy-3-indolylmethyl	Tryptophan
1MOI3M	4-methoxy-3-indolylmethyl	Tryptophan

Table S2 Univariate estimates of the effect of genotype on glucosinolate concentration

gene	Compound	Mean HET	Mean WT	P-value
Cyp79f1	3MSOP	0.173 (0.009)	0.266 (0.009)	$2.10*10^{-17}$
	4MSOB	1.580 (0.092)	1.892 (0.096)	$1.14*10^{-6}$
	5MSOP	0.068 (0.004)	0.056 (0.004)	0.053
	6MSOH	0.325 (0.015)	0.202 (0.016)	$1.80*10^{-7}$
	I3M	7.904 (0.341)	6.391 (0.358)	0.005
	4OHI3M	0.793 (0.034)	0.731 (0.036)	0.118
	1MOI3M	2.049 (0.112)	1.703 (0.118)	0.031
Cyp83a1	3MSOP	0.250 (0.011)	0.266 (0.009)	0.594
	4MSOB	1.910 (0.108)	1.892 (0.085)	0.124
	5MSOP	0.047 (0.005)	0.056 (0.004)	0.157
	6MSOH	0.176 (0.013)	0.202 (0.010)	0.475
	I3M	5.966 (0.387)	6.391 (0.303)	0.683
	4OHI3M	0.631 (0.041)	0.731 (0.032)	0.129
	1MOI3M	1.721 (0.131)	1.703 (0.102)	0.895
Sur1	3MSOP	0.307 (0.013)	0.266 (0.009)	0.001
	4MSOB	2.100 (0.124)	1.892 (0.089)	0.029
	5MSOP	0.057 (0.005)	0.056 (0.004)	0.737
	6MSOH	0.201 (0.016)	0.202 (0.012)	0.976
	I3M	5.870 (0.419)	6.391 (0.301)	0.660
	4OHI3M	0.725 (0.050)	0.731 (0.036)	0.915
	1MOI3M	1.594 (0.158)	1.703 (0.113)	0.018

Means (μmol/g) and standard errors are reported for untransformed data, while P-values are estimated from the log-transformed data. Standard errors are shown in parentheses.

Table S3 Univariate estimates of the effect of environmental treatments on glucosinolate concentration.

Treatment	Compound	Mean Untreated	Mean Treated	P-value
Water deprivation	3MSOP	0.238 (0.007)	0.238 (0.007)	0.880
	4MSOB	1.802 (0.070)	1.848 (0.072)	0.045
	5MSOP	0.060 (0.003)	0.058 (0.003)	0.526
	6MSOH	0.223 (0.011)	0.256 (0.012)	0.003
	I3M	6.443 (0.256)	7.085 (0.264)	0.019
	4OHI3M	0.832 (0.026)	0.629 (0.026)	$5.10x10^{-7}$
	1MOI3M	1.676 (0.092)	1.952 (0.095)	0.081
Crushing	3MSOP	0.223 (0.007)	0.253 (0.007)	0.077
	4MSOB	1.636 (0.069)	2.031 (0.072)	0.005
	5MSOP	0.052 (0.003)	0.066 (0.003)	0.800
	6MSOH	0.221 (0.011)	0.258 (0.012)	0.566
	I3M	6.093 (0.252)	7.482 (0.264)	$4.78x10^{-5}$
	4OHI3M	0.709 (0.026)	0.760 (0.027)	0.671
	1MOI3M	1.939 (0.092)	1.668 (0.096)	0.004
Soil nutrient deprivation	3MSOP	0.240 (0.007)	0.236 (0.007)	0.653
	4MSOB	1.975 (0.071)	1.676 (0.070)	0.001
	5MSOP	0.062 (0.003)	0.056 (0.003)	0.081
	6MSOH	0.254 (0.011)	0.224 (0.011)	0.063
	I3M	7.580 (0.258)	5.945 (0.255)	$1.07x10^{-5}$
	4OHI3M	0.661 (0.026)	0.805 (0.026)	$1.27x10^{-5}$
	1MOI3M	1.837 (0.094)	1.783 (0.094)	0.868
MeJA	3MSOP	0.242 (0.007)	0.233 (0.007)	0.596
	4MSOB	1.874 (0.070)	1.772 (0.072)	1.000
	5MSOP	0.051 (0.003)	0.067 (0.003)	0.260
	6MSOH	0.209 (0.011)	0.271 (0.011)	$1.71x10^{-7}$
	I3M	5.877 (0.252)	7.681 (0.259)	$5.18x10^{-12}$
	4OHI3M	0.769 (0.026)	0.696 (0.027)	2.819
	1MOI3M	1.427 (0.090)	2.215 (0.093)	$2.22x10^{-16}$

Means (μmol/g) and standard errors are reported for untransformed data, while P-values are estimated from the log-transformed data. Standard errors are shown in parentheses.
