# Supplementary material for: Flux Control in a Defense Pathway in Arabidopsis thaliana Is Robust to Environmental Perturbations and Controls Variation in Adaptive Traits
Source: G3 (Bethesda). 2015 Sep 10;5(11):2421–7. doi: 10.1534/g3.115.021816 (PMC4632061; doi:10.1534/g3.115.021816)
Supplement: Supporting Information [file supp_g3.115.021816_TableS1.pdf]

Table S1 Glucosinolate compounds examined in this study, with abbreviations and amino acid precursors.

Abbreviation	Glucosinolate	Amino acid precursor
3MSOP	3-methylsulfinylpropyl	Homomethionine
4MSOB	4-methylsulfinylbutyl	Dihomomethionine
5MSOP	5-methulsulfinylpentyl	Trihomomethionine
6MSOH	5-methylsulfinylhexyl	Tetrahomomethionine
I3M	Indolylmethyl	Tryptophan
4OHI3M	4-hydroxy-3-indolylmethyl	Tryptophan
1MOI3M	4-methoxy-3-indolylmethyl	Tryptophan
