# Supplementary material for: Flux Control in a Defense Pathway in Arabidopsis thaliana Is Robust to Environmental Perturbations and Controls Variation in Adaptive Traits
Source: G3 (Bethesda). 2015 Sep 10;5(11):2421–7. doi: 10.1534/g3.115.021816 (PMC4632061; doi:10.1534/g3.115.021816)
Supplement: Supporting Information [file supp_g3.115.021816_TableS2.pdf]

Table S2 Univariate estimates of the effect of genotype on glucosinolate concentration

gene	Compound	Mean HET	Mean WT	P-value
Cyp79f1	3MSOP	0.173 (0.009)	0.266 (0.009)	$2.10*10^{-17}$
	4MSOB	1.580 (0.092)	1.892 (0.096)	$1.14*10^{-6}$
	5MSOP	0.068 (0.004)	0.056 (0.004)	0.053
	6MSOH	0.325 (0.015)	0.202 (0.016)	$1.80*10^{-7}$
	I3M	7.904 (0.341)	6.391 (0.358)	0.005
	4OHI3M	0.793 (0.034)	0.731 (0.036)	0.118
	1MOI3M	2.049 (0.112)	1.703 (0.118)	0.031
Cyp83a1	3MSOP	0.250 (0.011)	0.266 (0.009)	0.594
	4MSOB	1.910 (0.108)	1.892 (0.085)	0.124
	5MSOP	0.047 (0.005)	0.056 (0.004)	0.157
	6MSOH	0.176 (0.013)	0.202 (0.010)	0.475
	I3M	5.966 (0.387)	6.391 (0.303)	0.683
	4OHI3M	0.631 (0.041)	0.731 (0.032)	0.129
	1MOI3M	1.721 (0.131)	1.703 (0.102)	0.895
Sur1	3MSOP	0.307 (0.013)	0.266 (0.009)	0.001
	4MSOB	2.100 (0.124)	1.892 (0.089)	0.029
	5MSOP	0.057 (0.005)	0.056 (0.004)	0.737
	6MSOH	0.201 (0.016)	0.202 (0.012)	0.976
	I3M	5.870 (0.419)	6.391 (0.301)	0.660
	4OHI3M	0.725 (0.050)	0.731 (0.036)	0.915
	1MOI3M	1.594 (0.158)	1.703 (0.113)	0.018

Means (µmol/g) and standard errors are reported for untransformed data, while P-values are estimated from the log-transformed data. Standard errors are shown in parentheses.
