# Supplementary material for: Flux Control in a Defense Pathway in Arabidopsis thaliana Is Robust to Environmental Perturbations and Controls Variation in Adaptive Traits
Source: G3 (Bethesda). 2015 Sep 10;5(11):2421–7. doi: 10.1534/g3.115.021816 (PMC4632061; doi:10.1534/g3.115.021816)
Supplement: Supporting Information [file supp_g3.115.021816_TableS3.pdf]

Table S3 Univariate estimates of the effect of environmental treatments on glucosinolate concentration.

Treatment	Compound	Mean Untreated	Mean Treated	P-value
Water deprivation	3MSOP	0.238 (0.007)	0.238 (0.007)	0.880
	4MSOB	1.802 (0.070)	1.848 (0.072)	0.045
	5MSOP	0.060 (0.003)	0.058 (0.003)	0.526
	6MSOH	0.223 (0.011)	0.256 (0.012)	0.003
	I3M	6.443 (0.256)	7.085 (0.264)	0.019
	4OHI3M	0.832 (0.026)	0.629 (0.026)	5.10×10^{-7}
	1MOI3M	1.676 (0.092)	1.952 (0.095)	0.081
Crushing	3MSOP	0.223 (0.007)	0.253 (0.007)	0.077
	4MSOB	1.636 (0.069)	2.031 (0.072)	0.005
	5MSOP	0.052 (0.003)	0.066 (0.003)	0.800
	6MSOH	0.221 (0.011)	0.258 (0.012)	0.566
	I3M	6.093 (0.252)	7.482 (0.264)	4.78×10^{-5}
	4OHI3M	0.709 (0.026)	0.760 (0.027)	0.671
	1MOI3M	1.939 (0.092)	1.668 (0.096)	0.004
Soil nutrient deprivation	3MSOP	0.240 (0.007)	0.236 (0.007)	0.653
	4MSOB	1.975 (0.071)	1.676 (0.070)	0.001
	5MSOP	0.062 (0.003)	0.056 (0.003)	0.081
	6MSOH	0.254 (0.011)	0.224 (0.011)	0.063
	I3M	7.580 (0.258)	5.945 (0.255)	1.07×10^{-5}
	4OHI3M	0.661 (0.026)	0.805 (0.026)	1.27×10^{-5}
	1MOI3M	1.837 (0.094)	1.783 (0.094)	0.868
MeJA	3MSOP	0.242 (0.007)	0.233 (0.007)	0.596
	4MSOB	1.874 (0.070)	1.772 (0.072)	1.000
	5MSOP	0.051 (0.003)	0.067 (0.003)	0.260
	6MSOH	0.209 (0.011)	0.271 (0.011)	1.71×10^{-7}
	I3M	5.877 (0.252)	7.681 (0.259)	5.18×10^{-12}
	4OHI3M	0.769 (0.026)	0.696 (0.027)	2.819
	1MOI3M	1.427 (0.090)	2.215 (0.093)	2.22×10^{-16}

Means (μmol/g) and standard errors are reported for untransformed data, while P-values are estimated from the log-transformed data. Standard errors are shown in parentheses.
